# Pregnancy-Triggered Vaso-Occlusive Crises in Hemoglobin SE Disease Complicated by Glucose-6-Phosphate Dehydrogenase Deficiency: A Case Report

**DOI:** 10.7759/cureus.102265

**Published:** 2026-01-25

**Authors:** Lubna M Alzahrani, Faten F Altassan, Wafa Althubaity

**Affiliations:** 1 Department of Family Medicine, Specialized Polyclinic, King Abdulaziz Medical City, National Guard Health Affairs, Jeddah, SAU; 2 College of Medicine, King Saud bin Abdulaziz University for Health Sciences, Jeddah, SAU

**Keywords:** diagnostic delay, g6pd deficiency, glucose-6-phosphate dehydrogenase deficiency, hbse, hemoglobin electrophoresis, hemoglobinopathy, hemoglobin se disease, pregnancy, sickle cell disease variant, vaso-occlusive crisis

## Abstract

Hemoglobin SE (HbSE) disease is a rare hemoglobinopathy that may be associated with significant clinical complications despite its traditional characterization as a mild condition, and concomitant glucose-6-phosphate dehydrogenase (G6PD) deficiency can further complicate diagnosis and management. We report the case of a 40-year-old Saudi woman with known G6PD deficiency who experienced recurrent vaso-occlusive crises triggered by pregnancy. She first presented postpartum in 2017 with severe joint pain, and initial hemoglobin electrophoresis suggested sickle cell trait (HbS: 27.1%, HbA: 67.9%). Six years later, during her fourth pregnancy complicated by gestational diabetes, she developed severe lower limb pain. Repeat hemoglobin electrophoresis demonstrated HbS of 66.3%, HbE of 27.1%, and HbA of 0%, confirming compound heterozygous HbSE disease. The delay in diagnosis was attributed to discrepant electrophoresis findings and a low index of clinical suspicion. This case underscores the importance of considering HbSE disease in patients with unexplained vaso-occlusive symptoms, particularly when pregnancy serves as a physiological stressor, and highlights that HbSE is not invariably benign. Awareness of the potential impact of coexisting G6PD deficiency on laboratory interpretation is essential, and comprehensive hemoglobin analysis, along with genetic counseling, is crucial for accurate diagnosis and appropriate family screening.

## Introduction

Sickle cell-hemoglobin E (HbSE) disease is a rare hemoglobinopathy resulting from compound heterozygosity for hemoglobin S (βS) and hemoglobin E (βE) mutations [1]. In Saudi Arabia, although the HbS gene is relatively prevalent compared with many other regions, the compound heterozygous state HbS/HbE remains poorly documented locally and is considered exceptionally rare worldwide. Hardy and Ragbeer previously described a Saudi family with both homozygous HbE and HbSE disease, further emphasizing the rarity of such double-heterozygous hemoglobin variants within the Saudi population [2]. Although HbSE disease has traditionally been classified as a mild sickle cell variant, accumulating evidence demonstrates marked clinical heterogeneity [3]. Patients with HbSE disease, particularly adults over 20 years of age, may develop serious multisystem complications, including hematologic manifestations such as chronic hemolytic anemia, splenic sequestration, and splenic infarction; vascular complications including vaso-occlusive crises, acute chest syndrome, and stroke; musculoskeletal involvement such as avascular necrosis and chronic pain syndromes; and systemic complications including cholelithiasis, renal impairment, and, in rare cases, premature death. These observations underscore the need for vigilant clinical monitoring, as HbSE disease cannot be assumed to follow a benign course [3]. Herein, we report a case of delayed diagnosis of HbSE disease in a 40-year-old Saudi woman with concurrent glucose-6-phosphate dehydrogenase deficiency, whose recurrent vaso-occlusive crises were precipitated by pregnancy. This case highlights the diagnostic challenges posed by discrepant hemoglobin electrophoresis findings and underscores pregnancy as an important physiological stressor in HbSE disease.

## Case presentation

A 40-year-old Saudi woman with a known diagnosis of glucose-6-phosphate dehydrogenase (G6PD) deficiency presented to the emergency department 10 days postpartum with acute bilateral knee pain and left shoulder pain of severe intensity (10/10), unresponsive to paracetamol. Her obstetric history was notable for three full-term spontaneous vaginal deliveries without antenatal or peripartum complications. Physical examination was unremarkable except for localized tenderness over both knees and the left shoulder. A knee radiograph was performed and revealed no abnormalities.

Initial laboratory investigations showed a hemoglobin level of 11.0 g/dL, hematocrit of 32.2%, white blood cell count of 9.2 × 10⁹/L, mean corpuscular volume of 68.5 fL, mean corpuscular hemoglobin concentration of 34.2%, and platelet count of 463 × 10⁹/L. A qualitative assay confirmed G6PD deficiency. The patient was managed conservatively in the emergency department with multiple doses of intravenous morphine, which provided temporary relief; however, her pain recurred shortly thereafter without a clear etiological diagnosis. She was subsequently referred to the adult hematology service and admitted for further evaluation. Peripheral blood smear demonstrated microcytic, hypochromic, and fragmented red blood cells. Hemoglobin electrophoresis was interpreted as consistent with sickle cell trait, showing HbA of 67.9% and HbS of 27.1%. She was treated with supportive care and analgesia and was discharged on meloxicam 15 mg and tramadol 50 mg as needed for pain, with advice to follow up in two weeks; however, she did not attend the scheduled appointment.

Several years later, the patient became pregnant again and was gravida 4, para 3, at 38 weeks’ gestation. Unlike her previous pregnancies, this pregnancy was complicated by gestational diabetes mellitus. She was admitted for delivery and reported acute lower back and right lower limb pain, predominantly involving the thigh and radiating to the foot. The pain had begun two days before delivery while she was asleep, initially localized to the right inguinal region and later radiating to the thigh and knee with an electric, burning quality. Postpartum, the pain extended from the knee to the calf, resulting in difficulty walking and significant impairment of daily activities. The pain was continuous, not related to activity or time of day, and was partially relieved by hip and knee flexion. She also recalled a similar episode during a prior postpartum period that had responded to diclofenac.

On physical examination, there was tenderness over the right mid-thigh and knee, with pain distributed along the L3-S2 dermatomes. Motor strength was preserved, and there were no clinical signs of deep vein thrombosis. Dorsiflexion, plantar flexion, and knee range of motion were intact but limited by pain. Laboratory evaluation revealed a hemoglobin level of 10.5 g/dL, a mildly elevated reticulocyte count of 4%, and a total bilirubin level of 52.5 µmol/L (indirect: 30.0 µmol/L, direct: 22.5 µmol/L). Hemoglobin electrophoresis demonstrated HbA of 0%, HbA₂ of 5.5%, HbF of 1.1%, HbS of 66.3%, and HbE of 27.1%, suggestive of a compound heterozygous state for HbS and HbE. Hematology consultation recommended repeat hemoglobin electrophoresis. The differential diagnosis included vaso-occlusive crisis, neuropathic pain, and G6PD-related hemolysis. Although mild hemolysis was evident, hemoglobin levels remained stable. The discrepancy between the earlier and later electrophoresis findings raised diagnostic uncertainty, possibly related to prior transfusion; however, the patient denied any history of blood transfusion.

The patient was managed with meperidine 100 mg every four hours and acetaminophen 1,000 mg for pain control. Neurology consultation suggested that the presentation was consistent with possible referred neuropathic pain and recommended outpatient nerve conduction studies. The patient’s pain resolved completely during hospitalization; however, she did not attend the scheduled outpatient follow-up. At routine postpartum follow-up, glycated hemoglobin (HbA1c) testing could not be reported due to interference from a hemoglobin variant. Repeat hemoglobin electrophoresis subsequently confirmed compound heterozygous hemoglobin SE disease (Table 1). The patient was referred to hematology for ongoing management and genetic counseling.

**Table 1:** Hemoglobin electrophoresis results “-” indicates parameters that were not measured in the initial electrophoresis study and reflect a testing limitation rather than true absence of the corresponding hemoglobin fractions.

Parameter	Initial study	Subsequent study	Confirmatory study
HbA	67.9%	0.0%	0.0%
HbS	27.1%	66.3%	66.2%
HbE	-	27.1%	27.8%
HbA₂	-	5.5%	5.0%
HbF	-	1.1%	1.0%

## Discussion

This case illustrates several important clinical lessons regarding HbSE disease. First, it demonstrates that HbSE is not invariably benign, challenging the traditional classification of this hemoglobinopathy as a mild variant [1,3]. Our patient experienced severe vaso-occlusive crises requiring hospitalization and parenteral analgesia, consistent with systematic reviews indicating that HbSE can present with complications comparable to those seen in other sickle cell variants [1]. Second, the prolonged interval between the initial presentation and definitive diagnosis highlights the diagnostic challenges inherent to compound heterozygous hemoglobinopathies, particularly when complicated by concurrent conditions such as G6PD deficiency.

The discrepant hemoglobin electrophoresis findings, initially showing HbA of 67.9% and HbS of 27.1%, and later demonstrating HbA of 0%, HbS of 66.3%, and HbE of 27.1%, warrant careful consideration. Although the patient denied any history of blood transfusion, the presence of HbA in the initial study raises the possibility of unrecognized transfusion, laboratory error, or sample misidentification. Coexisting G6PD deficiency may further complicate interpretation, as episodic hemolysis can alter the relative proportions of hemoglobin fractions detected on electrophoresis [4-7]. In female patients, lyonization resulting from X chromosome inactivation in G6PD deficiency can lead to variable degrees of hemolysis, potentially contributing to fluctuations in electrophoretic patterns. These considerations underscore the importance of repeat testing when clinical findings and laboratory results are discordant, as well as the role of confirmatory molecular testing, such as β-globin gene sequencing, in ambiguous cases [7,8].

Pregnancy appeared to trigger or exacerbate vaso-occlusive crises in this patient. Although her earlier pregnancies were uncomplicated, she developed severe postpartum joint pain following one pregnancy and significant lower limb pain during a subsequent pregnancy, which was further complicated by gestational diabetes mellitus. While data specifically addressing pregnancy-related risks in HbSE disease are limited, pregnancy is a well-recognized precipitant of complications in other sickle cell variants due to physiological stressors, including increased metabolic demand, hypercoagulability, relative hypoxia, and dehydration [1,5]. The additional metabolic burden imposed by gestational diabetes may have further increased the risk of vaso-occlusive events. These observations suggest that pregnant patients with HbSE disease may benefit from enhanced surveillance and proactive management strategies similar to those recommended for patients with HbSS or HbSC disease.

The clinical presentation during the later admission raised diagnostic uncertainty between neuropathic pain and vaso-occlusive crisis. The patient described burning, electric sensations with a dermatomal distribution (L3-S2), features suggestive of neuropathic pain. However, emerging evidence indicates that neuropathic pain is an underrecognized component of sickle cell disease, with a substantial proportion of adolescents and adults reporting neuropathic pain features [6]. Importantly, neuropathic pain and vaso-occlusive pain are not mutually exclusive; peripheral nerve ischemia during vaso-occlusive episodes can result in neuropathic pain that may persist beyond the acute crisis [6]. In this case, the temporal association with pregnancy and delivery, the presence of laboratory evidence of hemolysis, and the clinical response to analgesia support an underlying vaso-occlusive process with secondary neuropathic features. This highlights the need for comprehensive pain assessment in patients with sickle cell variants, recognizing that mixed pain mechanisms may influence therapeutic decisions.

Hemoglobinopathy screening should be considered in patients presenting with unexplained vaso-occlusive symptoms, particularly in populations with high carrier frequencies of hemoglobin variants [7]. In this context, the presence of microcytosis (mean corpuscular volume of 68.5 fL) and microcytic anemia in the absence of iron deficiency should prompt evaluation with hemoglobin electrophoresis. Discrepant or inconclusive electrophoresis findings necessitate repeat testing and, when appropriate, molecular confirmation using high-performance liquid chromatography or DNA-based techniques [8]. Accurate diagnosis of HbSE disease has implications beyond individual patient management, facilitating family screening, genetic counseling, and informed reproductive planning, especially in regions with a high prevalence of hemoglobinopathies [7]. In this case, delayed diagnosis resulted in several years of unmonitored disease and missed opportunities for anticipatory guidance and preventive care.

## Conclusions

This case report highlights several important clinical considerations. First, HbSE disease, although traditionally regarded as a mild condition, can be associated with significant complications, including severe vaso-occlusive crises, particularly when precipitated by physiological stressors such as pregnancy. Second, diagnostic challenges are common in compound heterozygous hemoglobinopathies, especially in the presence of concurrent conditions such as G6PD deficiency, which may complicate the interpretation of hemoglobin electrophoresis results. Third, discrepant or incongruent laboratory findings should prompt repeat testing and consideration of molecular confirmation rather than being disregarded. Finally, pregnancy represents a high-risk period for patients with HbSE disease and warrants enhanced clinical surveillance and multidisciplinary management.

Clinicians should maintain a high index of suspicion for hemoglobinopathies in patients presenting with unexplained vaso-occlusive symptoms, microcytic anemia, or evidence of hemolysis, particularly in populations with high carrier frequencies. Early and accurate diagnosis facilitates appropriate management, family screening, and genetic counseling, ultimately improving patient outcomes and reducing the risk of preventable complications. This case underscores the importance of comprehensive hemoglobin analysis and increased clinical awareness of HbSE disease.
